# Light color importance for circadian entrainment in a diurnal (*Octodon degus)* and a nocturnal (*Rattus norvegicus)* rodent

**DOI:** 10.1038/s41598-017-08691-7

**Published:** 2017-08-18

**Authors:** Maria Angeles Bonmati-Carrion, Beatriz Baño-Otalora, Juan Antonio Madrid, Maria Angeles Rol

**Affiliations:** 1 Chronobiology Laboratory, Department of Physiology, University of Murcia, IMIB-Arrixaca. CIBERFES, 30100 Espinardo, Murcia Spain; 2 Surrey Sleep Research Centre, School of Biosciences and Medicine, Faculty of Health and Medical Sciences, University of Surrey, Guildford, United Kingdom

## Abstract

The central circadian pacemaker (Suprachiasmatic Nuclei, SCN) maintains the phase relationship with the external world thanks to the light/dark cycle. Light intensity, spectra, and timing are important for SCN synchronisation. Exposure to blue-light at night leads to circadian misalignment that could be avoided by using less circadian-disruptive wavelengths. This study tests the capacity of a diurnal *Octodon degus* and nocturnal *Rattus norvegicus* to synchronise to different nocturnal lights. Animals were subjected to combined red-green-blue lights (RGB) during the day and to: darkness; red light (R); combined red-green LED (RG) lights; and combined red-green-violet LED (RGV) lights during the night. Activity rhythms free-ran in rats under a RGB:RG cycle and became arrhythmic under RGB:RGV. Degus remained synchronised, despite the fact that day and night-time lighting systems differed only in spectra, but not in intensity. For degus SCN c-Fos activation by light was stronger with RGB-light than with RGV. This could be relevant for developing lighting that reduces the disruptive effects of nocturnal light in humans, without compromising chromaticity.

## Introduction

The circadian timing system, a hierarchically organised network of structures responsible for generating ~24 h or daily rhythms, is driven in mammals by a circadian pacemaker located in the suprachiasmatic nuclei (SCN) of the hypothalamus. It allows organisms to adjust their physiology and behaviour and anticipate daily environmental changes, instead of merely reacting to them. The main input to keep the circadian system entrained to the external world is the light/dark cycle^1^. Light enters the circadian pacemaker through the eyes, and this pathway starts in a particular type of retinal ganglion cells (ipRGCs) containing melanopsin, which makes them intrinsically photosensitive^2^. These cells are directly excited by blue light (their peak of sensitivity has been demonstrated at 480 nm, although they are also activated by other wavelengths)^3^, and send the light irradiance information to the SCN through the retinohypothalamic tract (RHT) (intrinsic pathway)^4^. They also seem to be implicated in the pathways driving the arousal and sleep-promoting effects of blue and green light, respectively, that have recently been found in mice^5^. In addition, ipRGCs receive rod and cone inputs^6–8^, which constitute the extrinsic pathway. The discovery of melanopsin in some ganglion cells^2, 9^ and the knowledge of how light activates retinal photoreceptors^10^, together with the recently discovered subset of colour-sensitive SCN neurons^11^, demonstrate that not only light intensity and timing, but also its spectrum must be considered in order to keep the biological clock properly synchronised.

Currently, in developed countries, nights are excessively and inappropriately illuminated (*light at night*), whereas daytime hours are mainly spent indoors, and thus people are exposed to much lower and higher light intensities during the day and at night, respectively, than under natural conditions^12–16^. In spite of the positive impact of electricity, we pay a price for easy access to light during the night: the disorganisation of our circadian system or chronodisruption (CD), including perturbations in the melatonin rhythm^1, 17, 18^, a hormone with multiple physiological functions that also resets the SCN^19^ (for a review, see ref. 20).

CD can be defined as a relevant disturbance of the internal temporal order of physiological, biochemical and behavioural circadian rhythms, as well as a disruption of the physiological nexus between internal and external times^21^. This repeated disruption of the circadian system has been associated with several health impairments in humans^18, 22^, such as metabolic syndrome^23, 24^, cardiovascular diseases^25^, cognitive impairments^26^ and a higher incidence of breast cancer^27, 28^, among others.

Importantly, not all light wavelengths are equally chronodisruptive throughout the day. Along these lines, blue light (wavelengths from 460 to 480 nm^29^), is particularly beneficial during the daytime (e.g. it has been reported to have beneficial effects on well-being, cognitive performance and circadian status^30, 31^). However, it seems to be more disruptive at night, when it induces the strongest melatonin suppression^32^. Thus, the development of lighting systems that preserve synchronisation between external and internal timing could reduce health risks associated with CD. Since purple light (420 nm wavelength) is significantly weaker than 460-nm light in terms of melatonin suppression^33^, replacing the blue part (460–480 nm) of the spectrum with shorter wavelengths in nocturnal light could constitute an alternative in order to maintain synchronisation whilst preserving visual chromaticity.

Most previous studies evaluating the effects of different lighting conditions have been performed using nocturnal laboratory rodents, such as mice or rats. These studies have provided important insight into the effects of light on the circadian system. However, studies in diurnal animal models, whose circadian system and proportions of retinal photoreceptors are more similar to those of humans, are also necessary in order to evaluate the chronobiological effect of lights used during the night. This is important, especially considering that colour discrimination has been described as an influential regulator of SCN activity^11^. In recent years, the *Octodon degus*, a rodent from South America, has become a popular model in the fields of chronobiology and ageing. This animal exhibits a diurnal pattern of activity in its natural environment and in the laboratory, with peaks of general activity occurring in the morning and/or late afternoon^34^. In addition to the existence of downstream differences in how the SCN output signal is processed, differences associated with specific characteristics of the visual systems in nocturnal and diurnal species have been shown, such as higher proportion of cones and M1 ipRGCs in diurnal animals^35^. Consequently, it could be hypothesised that lights with the same spectral composition could be differently interpreted by the circadian system of diurnal and nocturnal rodent models in accordance with their different capacity to perceive colour, but this possibility has not been tested to date.

Thus the aim of this study was to evaluate whether modulating light spectral composition by replacing the blue part of the RGB spectrum with shorter wavelengths (RGV) can differentially entrain the circadian system of a diurnal (*Octodon degus*) and a nocturnal rodent model, the albino rat Sprague-Dawley (*Rattus norvegicus*) and, consequently, theoretically be used to synthesise a nocturnal light able to prevent the chronodisruptive effects of light at night, whilst maintaining a balanced chromatic vision.

## Results

### Activity patterns under different lighting conditions at night

**Figure 1:**
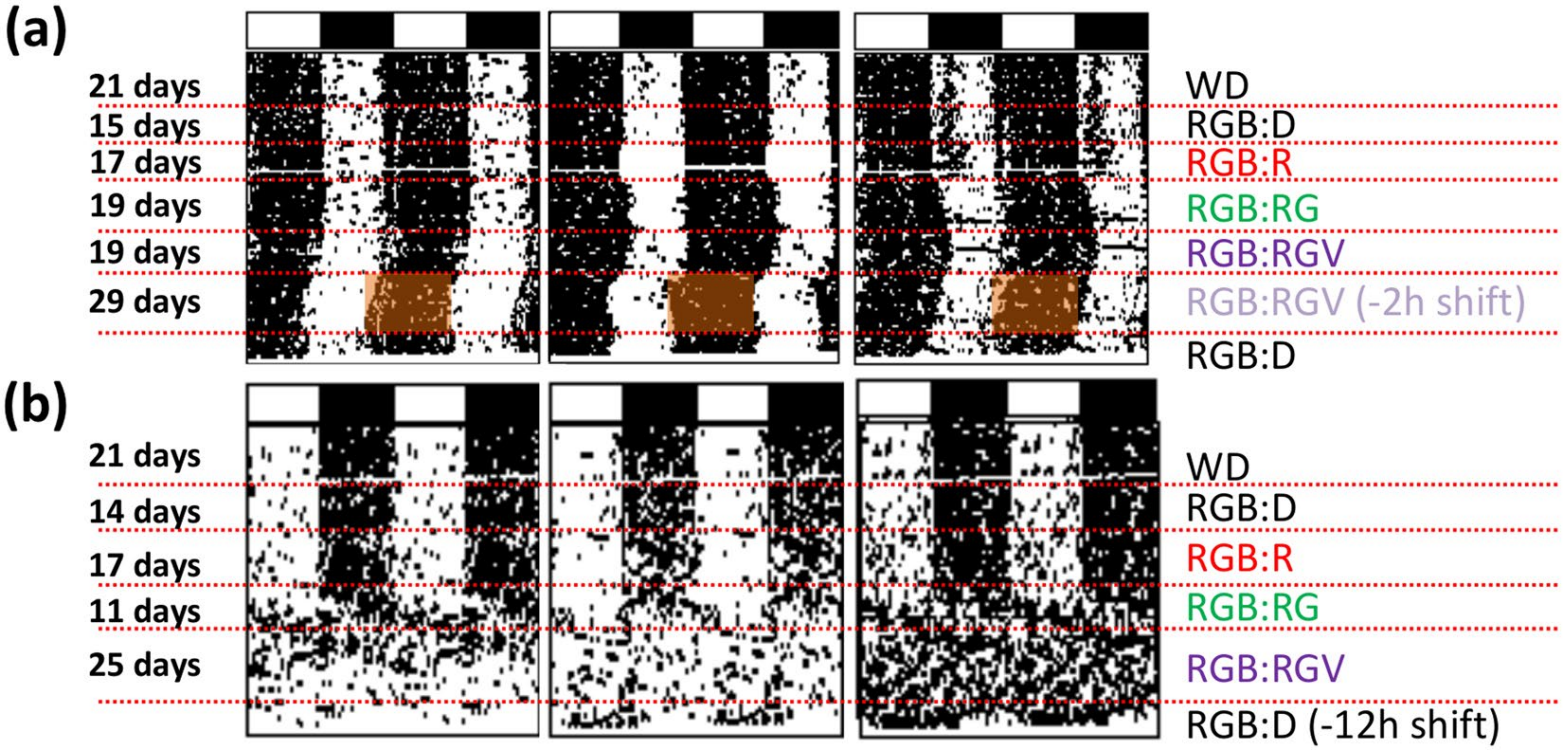
Actograms. Representative wheel running activity (WRA) double-plotted actograms from three degus (**a**) and three rats (**b**). White and black bars at the top of the actograms represent the daytime period (12 h) and night-time phase (12 h), respectively. Different light cycles throughout the experiment are shown on the right and indicated by dashed lines in the actograms. Animals were exposed to a combined red-green-blue LED (RGB) illumination for 12 h during the day. At night, they were subjected to different conditions: *i)* darkness (D); *ii)* red lights (R); *iii)* combined red-green LED (RG) lights; and *iv)* combined red-green-violet LED (RGV) lights. The (−2 h shift) indicates a 2 h phase advance of the RGB:RGV schedule (in degus) or a 12 h phase advance (−12 h shift) of the RGB:D cycle for rats. For more details on the lighting conditions, please see the methods section.

We first set out to determine whether the progressive introduction of different monochromatic lights during the night can affect wheel running activity (WRA) rhythms in degus and rats. To this end, the animals were subjected to different light cycles, beginning with W:D and RGB:D (white/RGB light during the day and darkness at night), and then a progressive and additive switching on of red (R), green (RG) and violet (RGV) light. The last condition, RGB:RGV, matched photon flux between day and night, and hence the only difference was the spectral composition. Figure 1 shows three representative WRA actograms for degus (1A) and rats (1B) under each light pattern. Most degus (88%) exhibited a diurnal chronotype, whilst all rats displayed typical nocturnal activity. As observed in the figure, both degus and rats remained synchronised with red light at night (RGB:R). When green light was incorporated at night (RGB:RG), some degus exhibited a slight phase delay, only to return to their previous entrainment phase a few days later. Some merely extended their locomotor activity period by delaying activity offset, whilst keeping the same activity onset. Interestingly, most (92%) also remained synchronised when violet light was lit during the night (RGB:RGV). The picture was quite different for rats (1B), who free-ran when green light was included at night and became arrhythmic when adding violet light (RGB:RGV). Thus, diurnal animals were able to remain synchronised under this nightlight, whilst nocturnal rats failed to do so.

**Figure 2:**
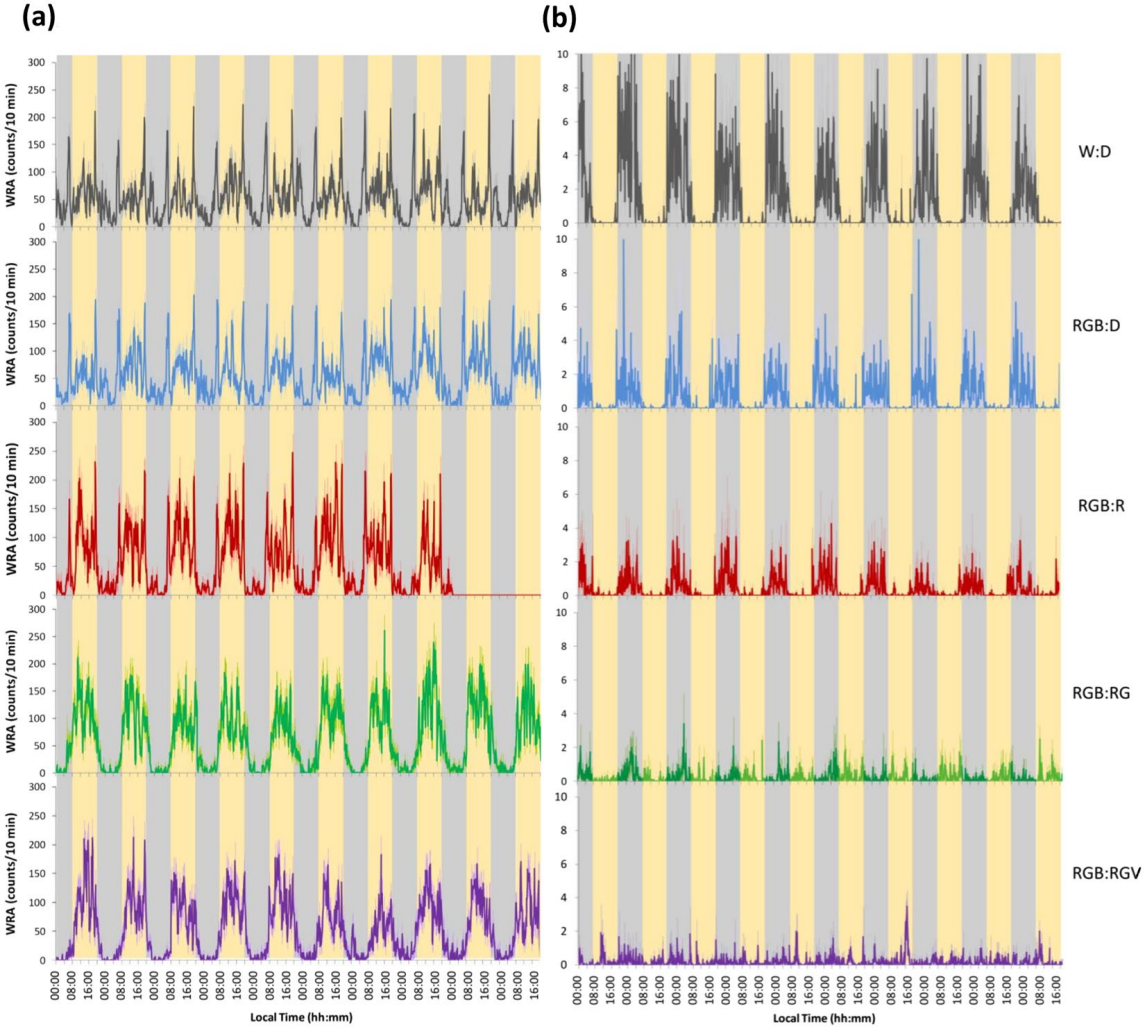
Average wheel running activity (WRA). Ten-day average WRA under the different light cycles in degus (3A, n = 27) and rats (3B, n = 12): W:D (grey line), RGB:D (blue line), RGB:R (red line), RGB:RG (green line) and RGB:RGV (purple line) (for more details, see the Materials and Methods section). Pale lines represent SEM. Grey and yellow columns indicate nocturnal and diurnal periods, respectively. For details on lighting conditions, see Fig. 9 legend. Two days from the RGB:R period are missing due to an error in the data acquisition system. Please note that the WRA scale for rats is not the same than that for degus.

**Figure 3:**
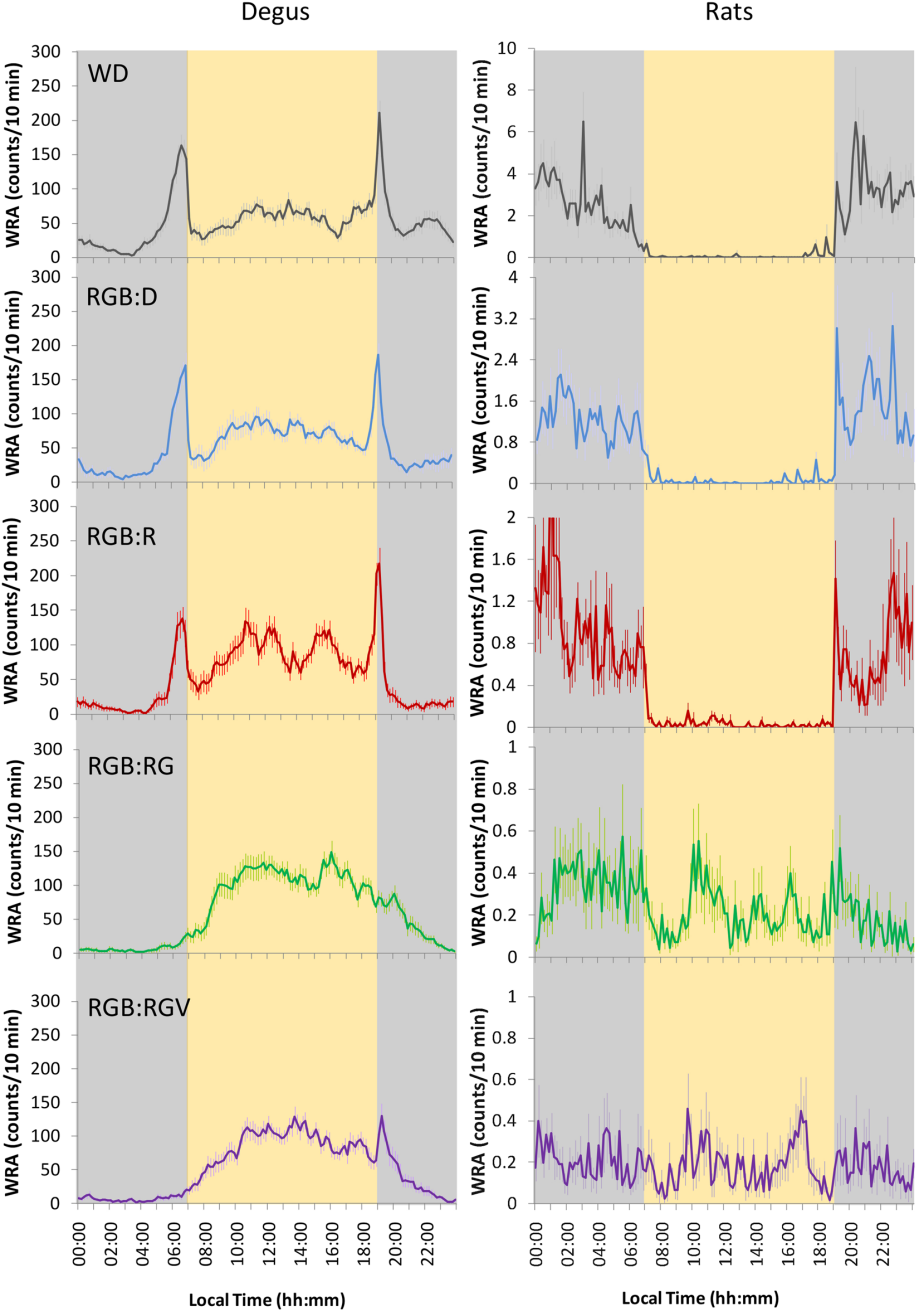
WRA mean waveforms. Mean waveforms of wheel running activity for degus (left panel, n = 27) and rats (right panel, n = 12) under the different lighting cycles: W:D (grey line), RGB:D (blue line), RGB:R (red line), RGB:RG (green line) and RGB:RGV (purple line) (for more details, see the Materials and Methods section). Pale lines represent the SEM. Grey and yellow columns indicate nocturnal and diurnal periods, respectively. For details on lighting conditions, see the Fig. 9 legend. Please note that the WRA scale for rats is not the same as for degus, or for the different light conditions.

When analysing the rest-activity pattern for each species, degus exhibited a predominantly diurnal pattern of WRA, with two crepuscular peaks after the onset of darkness/red nocturnal light and just before the diurnal light-onset (Fig. 2a). They maintained a similar activity/rest pattern throughout the study, even when nocturnal light was RGV (nightlight), the photon flux of which matched that of the diurnal RGB light (daylight). However, the typical peaks of motor activity around dusk and twilight^34^ disappeared when green light was introduced in the nocturnal period (RGB:RG). Interestingly, the peak around dawn tended to occur again when the violet light was lit (RGB:RGV) (Fig. 3, left panel), which could indicate the existence of two groups of oscillators.

Rats exhibited the expected WRA pattern, with activity concentrated in the nocturnal period under the W:D, RGB:D, and RGB:R light cycles (Fig. 2b). However, under RG in the nocturnal period, this pattern was attenuated, and WRA extended to the diurnal period (Fig. 3, right panel). When violet light was added (nocturnal light RGV), rats became completely arrhythmic. This difference in response to light between diurnal and nocturnal animals could be a consequence of the dissimilar capacity to perceive colours in accordance with their respective proportion in cones^36–40^ and ipRGC^41–45^, as detailed in the Discussion section.

**Figure 4:**
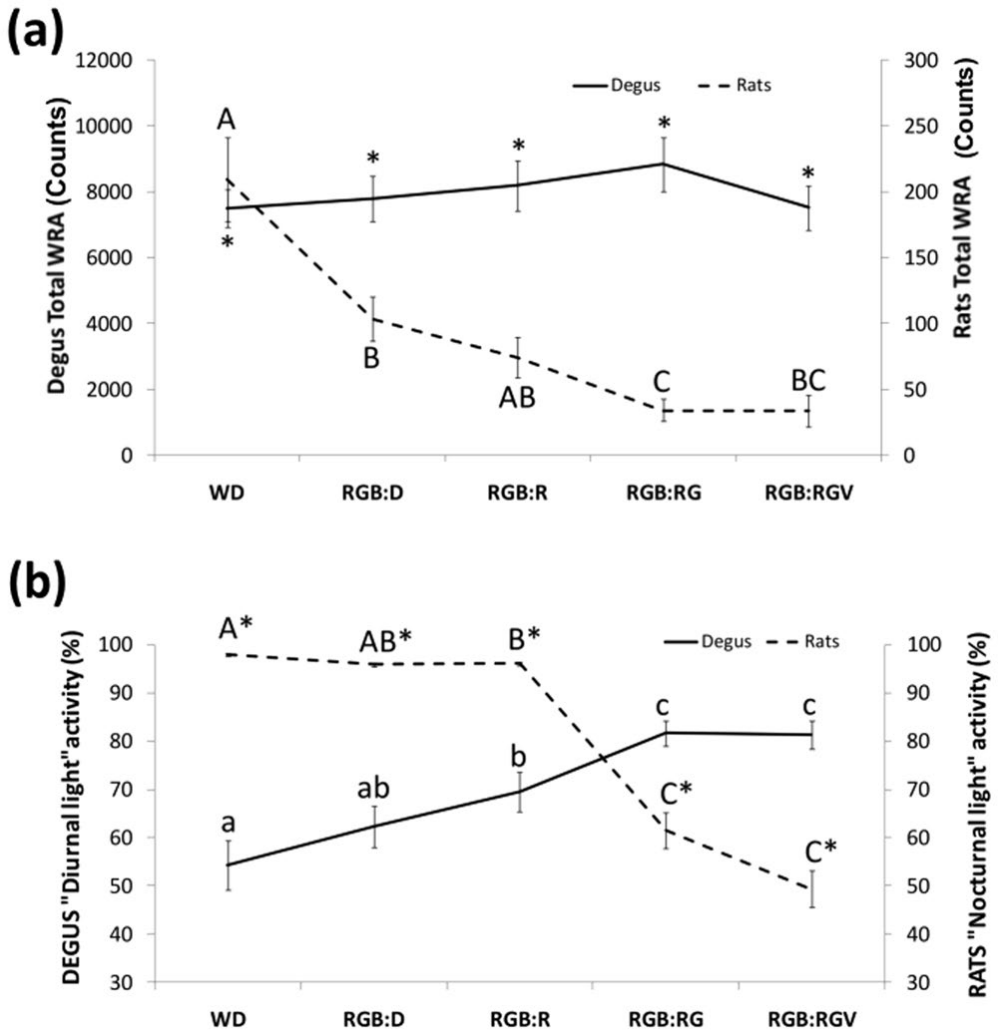
Total and day/night-time percentage of activity. (**a**) Total wheel running activity levels throughout the day (24 h) for degus (continuous line, n = 27) and rats (dashed line, n = 12). (**b**) Percentage of activity during the typical active period of degus (daytime) and rats (night-time). For more details, see the Materials and Methods section). Data are shown as the mean ± SEM. Different letters indicate statistically significant differences (repeated measures ANOVA, Bonferroni *post-hoc, p* < 0.05) between light conditions and * indicates statistically significant differences between degus and rats (Student’s t-test, *p* < 0.001).

Total WRA remained similar in degus throughout the different lighting cycles (Fig. 4a, repeated measures ANOVA, *p* > 0.05), whilst it progressively declined in rats in parallel with light at night turning on, with maximum values at WD (209.2 ± 31.8) and minimum values under RGB:RGV (33.9 ± 12.1) (ANOVA, Bonferroni *post-hoc, p* = 0.009). However, in degus the distribution of activity changed throughout the day (Fig. 4b). Indeed, their percentage of WRA during the diurnal phase (their predominant activity phase) was significantly higher when nocturnal lights were introduced, indicating that they were limiting all their activity to the daytime (repeated measures ANOVA, Bonferroni *post-hoc*, *p* < 0.013). Moreover, all degus exhibiting a nocturnal behaviour at the beginning of the experiment inverted their activity pattern and became diurnal when lights were turned on at night. Rats, on the other hand, progressively decreased their WRA during the nocturnal phase (their normal activity phase) with R, RG and RGV (repeated measures ANOVA, *p* < 0.001, Bonferroni *post-hoc*, *p* < 0.007).

**Figure 5:**
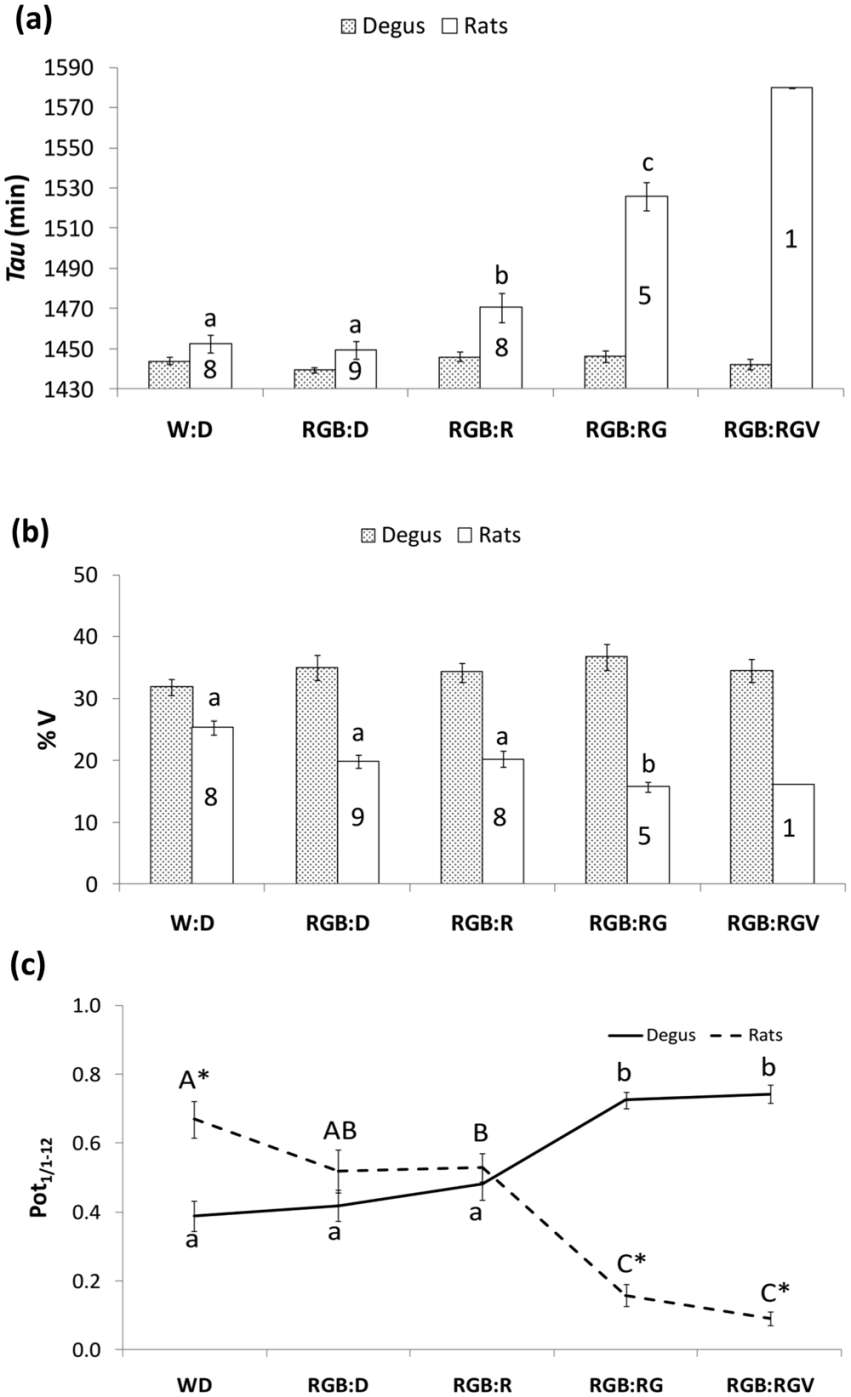
Period, percentage of variance and circadianity index. (**a**) Period (τ) and (**b**) %V of wheel running activity rhythm under different lighting cycles for degus (dotted bars) and rats (solid bars) (for more details, see the Materials and Methods section). Only animals showing significant periods were considered for statistical analysis (degus, n = 27; sample size for rats is indicated inside the bars). (**c**) Circadianity Index: Ratio (Pot_1/1-12_) between the first (Pot_1_) and the accumulative power of the first twelve harmonics (Pot_12_) for degus (continuous line) and rats (dashed line) under each lighting condition. Data are expressed as the mean ± SEM. Different letters indicate statistically significant differences between light conditions (repeated measures ANOVA, Bonferroni *post-hoc*, *p* < 0.05). *Indicates statistically significant differences between degus and rats (Student’s t-test, *p* < 0.001).

Degus were able to remain synchronised to a 24 h lighting cycle, regardless of the lighting conditions at night, as confirmed by the period (*Tau* parameter) (Fig. 5a). However, WRA period for rats was longer than 24 h with nocturnal lighting, increasing from 1452.22 ± 4.53 min (W:D, n = 8) to 1580 min (RGB:RGV, n = 1), and they became arrhythmic under this latter condition. Regarding the percentage of variance (Fig. 5b) explained by each period, we obtained similar values for degus (non-significant differences, *p* > 0.05) throughout all the stages, whilst in rats it decreased gradually from W:D (21.84 ± 1.81) to RGB:RGV (12.97 ± 0.38) (Bonferroni *post-hoc*, *p* < 0.024), which also confirmed the different response to the nocturnal light between both species.

The circadianity index (Pot_1/1-12_), consisting of the ratio between the power of the first Fourier harmonic and the accumulative power of the first twelve harmonics^46^, indicates the preponderance of the circadian over ultradian components. For degus, it was significantly higher under nocturnal green and violet lights (Fig. 5c) (Bonferroni *post-hoc*, *p* < 0.001). Rats again exhibited a behaviour that was opposite that of degus under nocturnal light, with lower circadianity indexes when green light was lit at night (Bonferroni *post-hoc*, *p* < 0.003). Therefore, our results point to this nocturnal light being able to strengthen the circadian component in degus, whilst decreasing it in rats, which underscores its deleterious effects on circadian entrainment in the latter.

**Figure 6:**
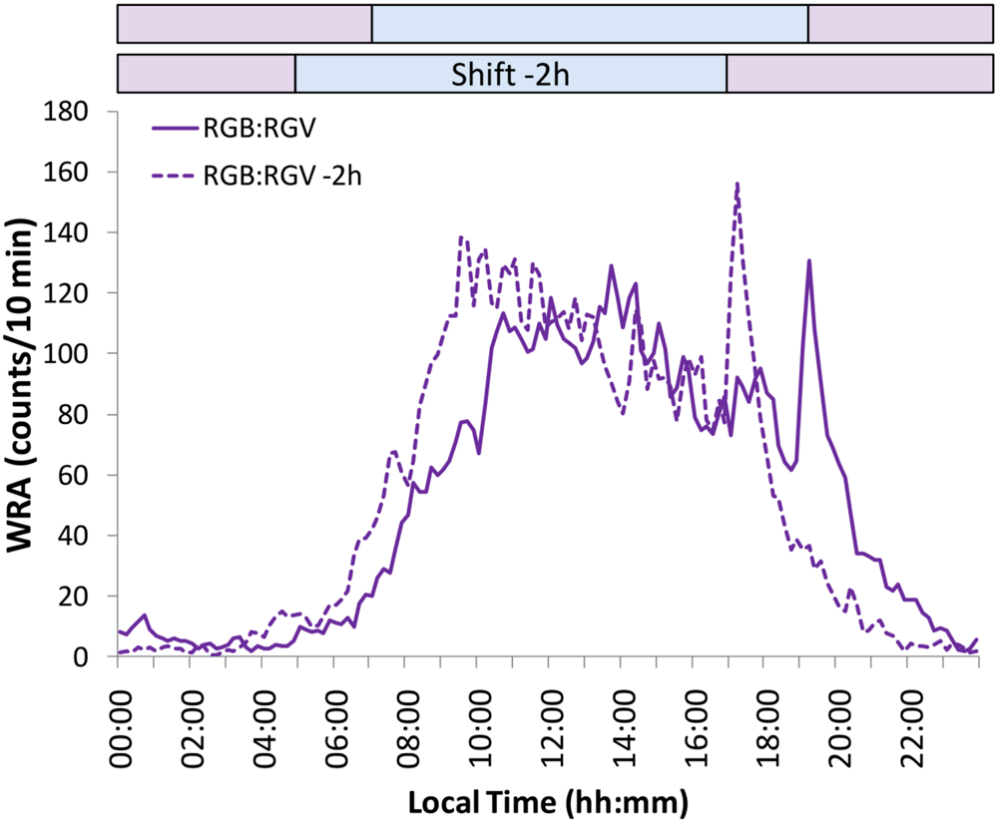
Phase shift challenge test. Wheel running activity mean waveform for degus (n = 25) under RGB:RGV light cycle, before (continuous line) and after (dashed line) a −2 h shift. Purple bars indicate the nocturnal period (RGV), whilst blue bars indicate the diurnal period (RGB) before and after the phase shift (Shift −2 h).

To demonstrate true entrainment in degus, and to exclude the possibility that *Tau* could match a 24 h-period by chance, we evaluated the capacity of degus to re-synchronise to a phase shift in the daylight/nightlight cycle. To this end, we performed a challenge test consisting of a 2-hour phase advance in the RGB:RGV cycle, in which light onset and offset were advanced two hours (daylight/RGB-on, at 06:00; daylight/RGB-off, at 18:00 h, local time). As can be observed in Fig. 6 (where mean waveforms calculated from the 10 days before and 10 days after the light cycle shift are represented), WRA shifted in accordance to light schedule. To assess this shift objectively, we calculated the phase marker M8 (see Materials and Methods), which significantly advanced from 14.69 ± 0.46 h to 13.23 ± 0.35 h (after the shift) (paired t-test, *p* < 0.001), which represents a phase advance of 1.46 ± 0.31 h. This rules out a possible artefact from period matching and confirmed that, indeed, the circadian system of degus was able to distinguish changes in spectral composition. In addition, this change in WRA phase took place after a few cycles of adaptation, which also permitted ruling out any possible masking effect.

### Motor activity responses to light pulses

In order to study possible masking by light, we subjected the animals to a 1-hour pulse of RGB or RGV light at CT16 (during their subjective night, after 28 hours of continuous darkness). Light at night causes positive masking in diurnal animals (activity increase), and negative in nocturnal animals (decrease in their activity).

**Figure 7:**
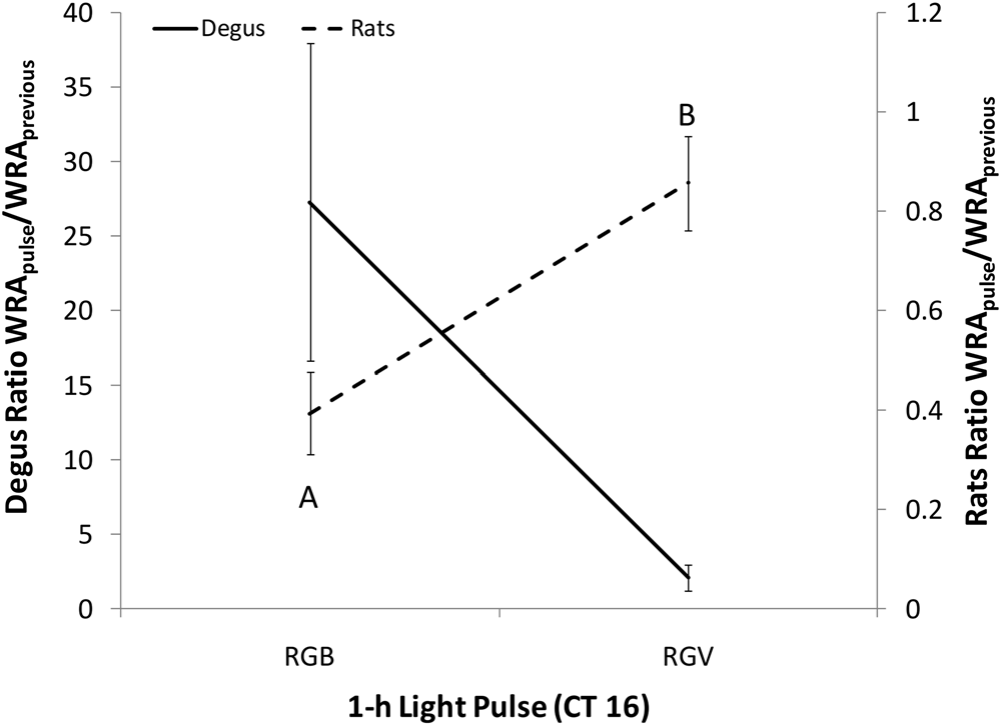
Acute effects of light on WRA. Ratio between wheel running activity (WRA) performed during the 1-hour light pulse (RGB or RGV) at CT16 and WRA performed the previous day at the same time. The left axis indicates the ratio for degus (continuous line, n = 8 for each light pulse) and the right axis indicates the ratio for rats (dashed line, n = 4 for each light pulse). Data are expressed as the mean ± SEM. Different letters indicate statistically significant differences (Student’s t-test, *p* < 0.05).

Under these conditions, the degus’ WRA (Fig. 7) increased by a factor of 27.29 ± 10.66 over the WRA performed the previous day at the same time, whilst the RGV 1-hour pulse only produced an increase that was 2.06 ± 0.85 times the previous day’s WRA levels (independent Student’s t-test, *p* = 0.077). In rats, the ratio was lower under RGB (0.39 ± 0.08 times the previous day’s WRA) than under RGV (0.86 ± 0.09) pulses (independent Student’s t-test, *p* = 0.011), indicating that the RGB pulse inhibited rat activity more than the RGV pulse did.

### Effects of different lighting conditions on melatonin levels and SCN activation

Melatonin is an important mediator in the circadian system; it is considered the *gold standard* phase marker, and also a good marker to evaluate the disruptive effects of light in humans. However, melatonin does not appear to be such good marker in degus^47, 48^. Plasma melatonin levels were quantified under the different lighting cycles (RGB:RGV), and also after the RGB and RGV light pulses, in order to determine possible suppression by light exposure during the night, since previous studies in rats showed significant protection of the melatonin nocturnal increase when short wavelengths (<480 nm) are filtered^49^. In our study, the plasma melatonin concentration was evaluated during the RGB:RGV light cycle, more specifically, at the RGB condition midpoint (thus, “midday”) and the RGV lighting midpoint (thus, “midnight”). In degus, it yielded 52.1 ± 6.85 and 53.8 ± 4.81 pg/mL for midday and midnight, respectively (n = 12), and 47.1 ± 6.98 and 58.1 ± 15.99 pg/mL, respectively, for rats (n = 6). However, in none of the cases were the differences statistically significant (Student’s t-test, *p* > 0.05).

Plasma melatonin concentration after 1-hour light pulses was not significantly altered in either degus (86.60 ± 21.99 for RGB pulse *vs*. 55.50 ± 9.99 pg/mL for RGV) or rats (80.20 ± 32.01 for RGB pulse *vs*. 54.3 ± 14.38 for RGV) as compared to controls maintained in darkness (62.60 ± 6.31 and 80.00 ± 22.07, respectively) (*p > *0.05).

**Figure 8:**
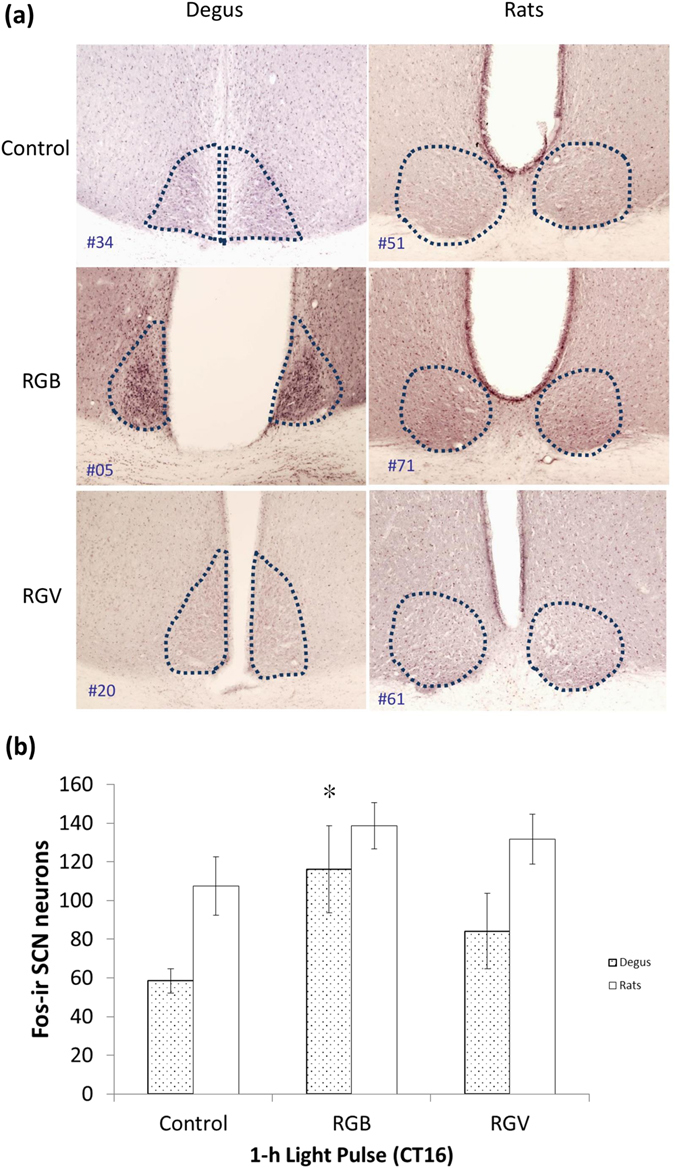
Effect of light on the suprachiasmatic nuclei. (**a**) Representative photomicrographs showing Fos-immunoreactivity in the suprachiasmatic nuclei (SCN) of degus (left panel) and rats (right panel) under each experimental condition: control animals, which received no light pulse (darkness), and after 1-hour light pulse at CT16 with RGB or RGV lights. The SCN area is delimited by dotted lines. (**b**) Quantification of Fos-immunoreactive neurons (counted on the left part of the SCN) in the control group (no light pulses, n = 9 degus; 4 rats), the RGB group (pulsed for 1 h with RGB light at CT16, n = 7 degus; 4 rats) and the RGV group (pulsed for 1 h with RGV light at CT16, n = 7 degus; 4 rats). Data are expressed as the mean ± SEM. *Indicates significant differences compared to control degus (Kruskal-Wallis, *p* = 0.030; Mann-Whitney U test, *p* = 0.034).

In order to evaluate the direct effect of both lights (RGB, our daylight; and RGV, our nightlight) on the central circadian pacemaker, we assessed SCN neuronal activation through c-Fos expression, which indicates neuronal activation. This tended to be higher in both degus and rats subjected to the RGB light pulse than in animals subjected to the RGV pulse or darkness (control), as can be appreciated in terms of the number of immunoreactive neurons shown in Fig. 8a and b. However, RGB light produced stronger effects than RGV light in the degus’ SCN (Mann-Whitney U test, *p* = 0.034), but not in the rats’ SCN.

## Discussion

In this study, we evaluated the circadian system response to changes in light spectral composition (specifically, by replacing the blue part of the spectrum with purple light), in both a diurnal (*Octodon degus*) and a nocturnal species (*Rattus norvegicus*), demonstrating how colour information affects the entrainment capacity differently, a condition that can be exploited to generate a synthetic light for preventing the chronodisruptive effects of light at night.

The activity-rest pattern, one of the main outputs of the circadian system^50^, showed that the night lighting we designed was able to keep degus synchronised to 24 h, whilst it failed to do so with the nocturnal rat. Degus maintained similar levels of total WRA under the different light conditions, as previously described with increasing intensities of continuous light^51^. They also kept their diurnal pattern, exhibiting an even higher percentage of WRA during the day when light at night was introduced, which is consistent with previous studies on diurnal rodents^52^. Furthermore, we maintained the same photon density for both phases, and even still, the motor activity pattern in degus was restricted to daytime (RGB). In our study, degus maintained their period (τ) under the different light cycles, contrary to what has been described in previous studies, in which degus exposed to light during the night lengthened their τ^51^.

However, rats, as nocturnal animals, decreased their total WRA when light components were progressively turned on at night, which is in agreement with previous studies^53^. For rats, both RG and RGV lights seem to be perceived by their circadian system as daylight, since they began to free run when green light was lit, and became arrhythmic when violet light was also present, as occurs under constant lighting conditions^54^. Furthermore, rats increased their τ as nocturnal lights were introduced, which is in accordance with Aschoff’s first rule^55–57^. Moreover, the number of rats with significant τ decreased, confirming that a high percentage of them became arrhythmic under nocturnal lighting, as described for constant light exposure in these animals^54^. In this respect, a recent study found that blue and green lights produce, arousal- and sleep-promoting effects, respectively, in mice, which means that ipRGCs are implicated in both pathways^5^. This phenomenon could explain the arrhythmic pattern that rats exhibited when green, but not blue, light was used during the night.

Although degus were able to keep their motor activity pattern synchronised to the daylight/nightlight cycle, they exhibited changes in their motor activity pattern in response to the nocturnal lights. Firstly, some of them presented a slight transient delay in their activity onset and offset or only in the onset, increasing their activity period (*alpha*), as has been previously described^51^. The interesting fact is that, in our study, they recovered their previous activity onset after a few days and that there was no clearly different effect after introducing the violet component, although the tendency was opposite. The latter observation could be due to the high light intensity used at the final stages (RGB:RG and RGB:RGV), which could result in increased *Tau* and in an elevation of the activity, which is similar to the free run behaviour previously observed in degus^51^.

Secondly, they lost their typical peaks of activity after the onset of darkness and just before the onset of light^34^. These two peaks were maintained with red light at night, but disappeared when green light was turned on, although the peak after the offset of daylight reappeared when violet was lit at night (RGV). It could be hypothesised that the two groups of oscillators (one for dawn and one for dusk) with a different degree of coupling between them, as previously described in degus^58, 59^, could present different sensitivity to light spectrum. This is also consistent with the recent discovery of colour-sensitive SCN neurons, which allow the animal to integrate not only the information about irradiance, but also the spectral composition of light, which is essential to track the time of the day^11^.

The fact that lights were progressively introduced at night could be a weakness of this study, since animals could have progressively adapted to the new irradiance. However, true entrainment for degus, excluding a *Tau* coinciding with 24 h, was demonstrated by phase advancing the RGB:RGV cycle, confirming that the circadian system of degus was indeed able to discriminate between diurnal and nocturnal lights when only spectrum, but not light intensity, was changed. In addition, the WRA peak associated with dusk also advanced by around two hours, indicating the high sensitivity of the degu’s SCN to blue-violet switch.

Although the presence of wheel running activity could influence the circadian system function, we chose this recording system because it only registers voluntary movements (reducing activity artefacts during the rest phase)^60^; also the positive feedback loop between physical exercise and the circadian pacemaker could facilitate the robustness of circadian rhythmicity in both species^61, 62^. It is also a recommendation to keep health and wellbeing of laboratory animals housed in cages^63^.

In our study, instead of eliminating short wavelengths by filters^49^, we chose to replace the most effective range for the circadian system (460–480 nm) with violet light (shorter wavelength), which has been reported to produce less melatonin suppression in humans than bluish lights^33^ and also produces less activation of the ipRGCs^10^. Surprisingly, melatonin concentration in degus during the daytime was different from that previously reported under LD conditions^47, 48^, whilst at CT16 (night-time), levels under RGB and RGV were similar to those obtained during the day, with the same occurring in rats. Other authors^49^ have reported higher nocturnal melatonin concentration in rats than in our case, but we must consider that, in this phase of our experiment, the rats were already arrhythmic. Furthermore, it has been described that rats exposed to long-term continuous light present detectable melatonin levels, but with no differences between day and night^64, 65^ or with peaks at random times, which are not associated with the manifestation of the motor activity^64^.

It has been previously reported that peak plasma melatonin levels may show marked variations among individuals, and also in the circadian time at which the maximum occurs. Although degus were synchronised to the nocturnal-diurnal light cycle, we cannot rule out the possibility that the melatonin rhythm could be differentially affected with respect to other circadian outputs, as has been suggested for rats^64^. Previous studies performed in our laboratory also showed that melatonin is not a good marker of the circadian system of the degu, since its rhythm can be phase shifted with locomotor activity^47, 48^ even under usual LD cycles.

As expected, c-Fos activation, which indicates neuronal activation in the SCN, tended to be higher under 1-hour pulses than under darkness (control) for both degus and rats, which is in agreement with previous studies^66^. The degu’s SCN light-sensitive cells have been reported to require much higher levels of light to induce a response^67^ than those of the rat, but the fact that RGB light was significantly more effective in degu’s SCN than RGV light could indicate a greater ability by degus to distinguish different light spectra.

The WRA masking produced by both daylight (RGB) and nightlight (RGV) was also different in the diurnal and nocturnal models, as previously described^68^. Rats, as nocturnal animals, generally become more active in response to darkness or low light levels (positive masking) and less active in response to light (negative masking), whilst diurnal animals, such as degus, generally exhibit the opposite response. In any case, the masking effect was more pronounced under RGB (daylight) than under RGV light pulses (nightlight), which also agrees with the fact that ipRGC (sensitive to blue light) are also implicated in masking^44, 69^. Again, these results could confirm that daylight, but not nightlight, is processed as true light input, activating the masking centres of the circadian system in both degus and rats.

Thus, the synthetic nightlight RGV seems to be processed by the degu’s circadian system as darkness because: i) a photon-matched RGB:RGV cycle is able to entrain its locomotor activity rhythm to this RGB “photophase”; ii) it responds, after several cycles of transition, to a phase advance in the RGB:RGV cycle; iii) SCN neurons are less activated by a RGV pulse than by a RGB pulse; iv) the positive masking of light on motor activity generated by an RGB pulse seems not to occur with a RGV pulse.

In addition to the existence of downstream differences in the way in which the output SCN signal is processed, differences associated with the visual systems in nocturnal and diurnal species should be considered^35^. For both degus and rats two cone mechanisms have been described, corresponding to S- (λ_max_~360 nm) and M-cones (λ_max_~500–510 nm), as well as a rod mechanism with a λ_max_~ of around 500 nm for degus^37^ and rats^70–73^. However, the distribution and percentage for each photoreceptor is different in diurnal and nocturnal species. In diurnal rodents, such as *O. degus* and *Arvicanthis ansorgei* ~30% of photoreceptors are cones^36, 37^, whilst the percentage for nocturnal rodents is much lower (2–3%)^38^. The difference becomes even more dramatic when considering albino nocturnal animals, such as in our case, since just 1% of the photoreceptors are cones^39^. These differences could be especially relevant, according to the recent discovery of a subset of colour-sensitive SCN neurons involved in tracking the spectral composition changes that occur throughout the day, which allow animals to integrate irradiance and colour information, a fundamental cue to estimate the time of day^11^. Indeed, electrophysiological recordings showed differences in the photic responsiveness of SCN neurons in degus compared to rats^74^. With regard to the five different ipRGC subtypes (M1-M5), it has been reported that M1, which innervates the SCN (the main pacemaker) exists in a higher percentage (74%) in diurnal species, such as *Arvicanthis ansorgei* ^44^ as compared to nocturnal (30–44%), such as mice^42, 43, 45^. Thus, the greater cone contingent and higher percentage of M1 ipRGCs in diurnal rodents would explain why rats are unable to entrain under the input contrast between RGB *vs*. RG and RGV, whilst degus are capable of doing so. The lower percentage of cones and ipRGC in our nocturnal model could be responsible for the lack of entrainment by rats to the RGB:RGV condition, although we cannot discard other differences in how the light signal is processed. Degus, however, at photoreceptor level, are able to differentiate light containing blue and violet^37^. Furthermore, different photoreceptor sensitivities in both species could underlie the different responses seen in both models. In addition, future studies analysing dose-response to monochromatic lights would confirm the spectral sensitivity in degus.

It is possible that rat photoreceptors were saturated at the final stage (as previously shown in mice^75^, so further studies with the same proportion of each light component but different total irradiance should be performed to confirm these differences in input and output light signal processing between both species. Thus, differential effects for day- and night-time light should be also studied in diurnal species with photopic vision, such as degus, especially when the conclusions are to be extrapolated to humans. Although albino animals have been traditionally used, and will probably continue being used for physiological investigations, they should be avoided in circadian studies. In addition, as demonstrated in this study, light spectrum changes are important not only for circadian entrainment, as they could also lead to other physiological alterations.

Our results provide additional evidence for the importance of colour in aligning the clock with the external time, and they suggest that replacing the blue component of light with violet could be a strategy to illuminate the night with minimal impairment of the circadian system in a diurnal species. However, further studies including nocturnal light that is lower in intensity than diurnal light and complementary techniques in humans (such as pupillometry) should be tested to progress towards achieving circadian-friendly lighting.

## Methods

### Animals and housing conditions

All experimental procedures were performed in accordance with the Principles of Animal Care and Spanish laws, and approved by Ethical Committee for Animal Research of the University of Murcia in compliance with the Spanish RD53/2013 Law. Twenty-seven *Octodon degus* (15 males and 12 females) between 13 and 17 months of age were obtained from the Animal Facilities at the University of Alicante (Spain), and twelve 4-month old adult male Sprague-Dawley (SD) rats were from the Animal Facilities at the University of Murcia (Spain). Considering that the life span of degus is around 7 years in captivity^76^ and rat’s is around 2.5 years, at the moment of starting the experiment, they were both adults of similar biological age, being namely, at 17 and 14% of their maximal life span.

All animals were individually housed in polycarbonate cages (Panlab S.L., Barcelona, Spain) equipped with exercise wheels (52 × 15 × 27 cm, length × height × width) in isolated chambers (“Chronolab”) with controlled relative humidity (60% ± 10%), ambient temperature (24.1 °C ± 0.3 °C), and photoperiod (12:12 diurnal light: darkness/nocturnal light, with daylight-ON from 08:00 to 20:00 h local time, except when phase advance was programmed). Animals were fed commercial rat chow (A04 rat-mouse maintenance Panlab, S.L., Barcelona, Spain) and drinking water *ad libitum*.

### Light exposure

**Figure 9:**
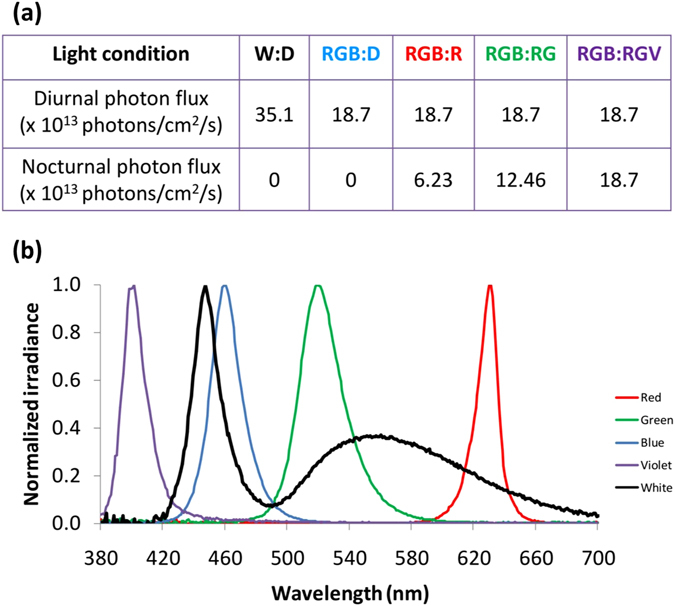
Photon fluxes and spectra for the different light conditions used. (**a**) Photon fluxes used for light at daytime (12 h): night-time (12 h): W:D (white LED:darkness), RGB:D (combined red, green and blue LEDs: darkness), RGB:R (combined RGB LEDs: red LED), RGB:RG (combination of RGB LEDs: red and green LEDs), RGB:RGV (combination of RGB LEDs: combination of red, green and violet LEDs). (**b**) Spectrum for the white LED and the monochromatic components of the different light conditions (red, green, blue and violet).

All the animals were exposed to different light cycles: two types of diurnal light were used, one provided by white (W) LED strings (at 35.1 × 10^13^ photons/cm^2^/s) and the other, our tested daylight (RGB), composed of red (peak at 630 nm), green (peak at 518 nm), and blue (peak at 459 nm) monochromatic 12 V LED strings (Ingebat, Valencia, Spain) at 18.7 × 10^13^ photons/cm^2^/s (Fig. 9). White LED was used to test whether the change to a composed RGB light could affect wheel running activity levels.

The nocturnal lights tested were red (R) at 6.23 × 10^13^ photons/cm^2^/s, red combined with green (RG), at 12.46 × 10^13^ photons/cm^2^/s, and red with green and violet (RGV, our proposed nightlight, with V peak at 401 nm), at 18.7 × 10^13^ photons/cm^2^/s (Fig. 9). Light conditions were scheduled by an electronic timer (Data Micro, Orbis, Madrid, Spain). Light irradiances were verified at cage level, using a calibrated radiometer (R203, Macam Photometrics Ltd., Livingston, Scotland). The irradiance was chosen based on methodological factors (in order to match photon fluxes as much as possible amongst conditions) and considering the levels used in the literature (refs 8, 11). The actual *in situ* λ_max_ at cage level was measured by a calibrated spectrometer (Ocean Optics BV, Dunedin, Florida, USA).

### Data Recording

Wheel-running activity (WRA) was recorded as wheel revolutions/10 min intervals, using a data acquisition system (Electronic Service at the University of Murcia, Spain), as previously described^48, 59^.

### Experimental design

**Figure 10:**
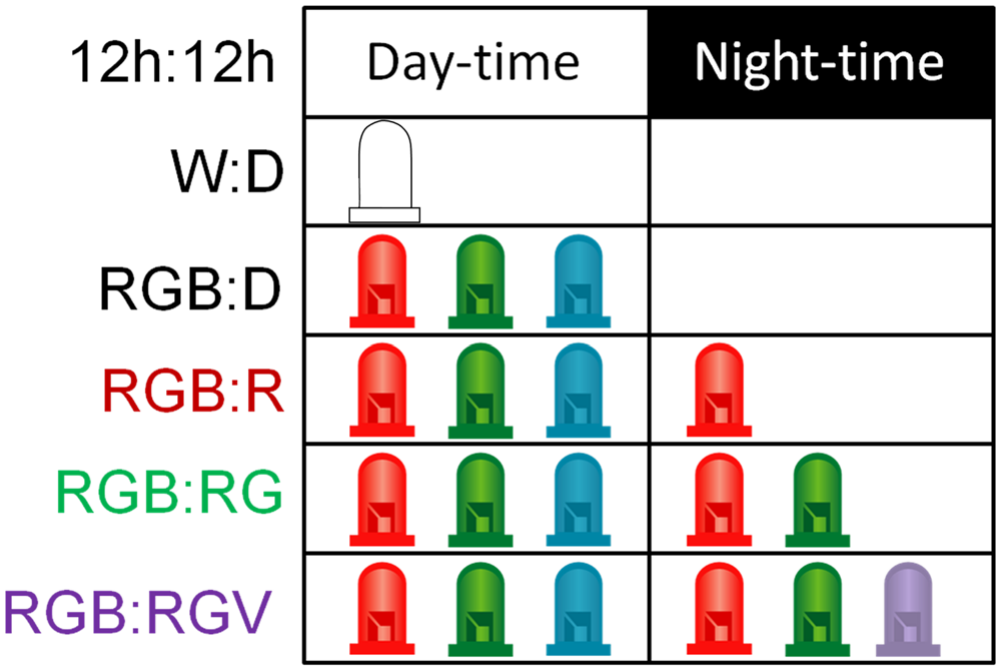
Experimental design. Animals (27 degus and 12 rats) were exposed to white or a combined red-green-blue LEDs (RGB) illumination for 12 h during daytime. At night, they were subjected to different conditions: *i)* darkness; *ii)* red light (R); *iii)* combined red-green LED (RG) lights; and *iv)* combined red-green-violet LED (RGV) lights.

Initially animals were kept with running wheels under a 12:12 white light: dark (W:D) cycle for 21 days. Then, the diurnal light was changed from W to RGB, maintaining darkness during the night (RGB:D). The animals were kept under this light cycle for two weeks. Afterwards, monochromatic lights were incorporated step by step into the nocturnal period, beginning with red (RGB:R) (17 days), then green (RGB:RG) (at least 11 days), and finally violet (RGB:RGV) light (19 days for degus and 25 days for rats) (Fig. 10). Red light was introduced in the first stage (although no L-cone system is found in either rats nor degus) due to the evidence previously found that red light affects circadian rhythms in rats^64, 77^.

Degus were then subjected to a 2-hour phase advance under a RGB:RGV schedule and they were maintained under this new schedule for 29 days. Animals were then returned to RGB:D lighting conditions the week before the experiment ended.

At the end of the experiment, the animals were released under constant darkness for 28 hours before receiving a 1-hour light pulse (RGB, RGV or control) during their subjective night (CT16, corresponding to 4 hours after activity offset for degus, and activity onset for rats). Light pulses were photon-matched with the previous light conditions and groups were sex-balanced.

### Wheel running activity analysis

WRA actograms, mean waveforms, periodograms and Fourier analysis parameters were calculated using software specifically designed for time series analysis (El Temps version 1.228; © Díez Noguera, University of Barcelona). To determine the significant periods of the WRA, Sokolove-Bushell periodograms with Bonferroni correction^78^ were performed for each animal during the five experimental stages (W:D, RGB:D, RGB:R, RGB:RG and RGB:RGV). This method allows us to identify different rhythmic components. The percentage of variance (%V) explained by each period was used as an indicator of the importance of each component. From the Fourier analysis, the ratio between the first (Pot_1_) and the accumulative power of the first twelve harmonics (Pot_1–12_) was calculated (Pot_1/1-12_) in order to obtain the circadianity index^46, 79^, a measure of the power of circadian over ultradian periods. The percentages of diurnal WRA for degus and nocturnal WRA for rats were also calculated. All these analyses were performed for 10-day periods, once animals had already been under each condition for 4 (degus) and 2 (rats) days.

To determine whether the degus were able to resynchronise to a 2 h phase shift on the diurnal (RGB)/ nocturnal (RGV) light cycle, the M8 non-parametric phase marker (the central timing of the eight consecutive hours with the highest WRA values) was calculated for 10 days prior to and following the phase advance.

Effects of the 1-hour pulses of different lights on WRA levels were expressed as the ratio between the averaged WRA during the 1 h light pulse and the averaged WRA on the previous day at the same time.

### Tissue Preparation

Venous blood samples were obtained from slightly anesthetised animals (fluothane 2.5% mixed with oxygen for induction and 1.5% for maintenance) through jugular venopuncture, as previously described^48^. Samples were collected in the middle of the day and in the middle of the night period from animals maintained under an RGB:RGV schedule.

At the end of the experiment, after 1-hour light pulses, all animals were deeply anesthetised using a solution of ketamine (100 mg/kg) (Imalgène 1000) and xylazine (4 mg/kg) (Xilagesic® 2%), and blood samples were obtained by cardiac puncture. Blood samples with Heparin 5% as an anticoagulant (Hospira®, Madrid, Spain) were immediately centrifuged at 2026 g for 15 min at 4 °C. The plasma obtained was stored frozen at −80 °C until quantification. This process was performed under dim red light, with light-tight hoods fitted over the animal’s heads to further reduce the possibility of light exposure during this procedure.

The animals were then transcardially perfused with 250 ml of 0.9% saline, followed by 500 ml of 4% paraformaldehyde in 0.1 M phosphate-buffered saline (PFAS; pH 7.4). Afterwards, the brains were carefully removed and post-fixed for further 24 h in PFAS, then transferred into 30% sucrose prepared in 0.1 M Phosphate Buffer (PB). After 48 h in sucrose, brains were fast-frozen in isopentane cooled with dry ice and stored at −80 °C until sectioning. Brains were sliced to a thickness of 40 µm on a freezing sledge microtome (Series 8000, Bright Instruments Ltd. Huntingdon, UK).

### Immunohistochemistry

Brain sections from different experimental groups were processed in parallel. Free-floating sections were washed for 5 × 30 min in 0.1 M PBS. To suppress endogenous peroxidase activity and reduce background staining, the sections were then incubated for 20 min in 1.5% hydrogen peroxide (Sigma, H1009, Poole, UK) prepared in 0.1 M PBS-TX (PBS containing 0.03% Triton X-100). The sections were then washed 4 × 10 min in 0.1 M PBS and pre-incubated for 1 h in a blocking solution containing 5% normal donkey serum (NDS, Jackson ImmunoResearch Laboratories, West Grove, PA, USA) in 0.1 M PBS-TX. Brain sections were then incubated at 4 °C for 48 h in anti-c-fos primary antibody (sc-52, rabbit polyclonal 1:5000; Santa Cruz Biotechnology, Santa Cruz CA, USA) containing 5% NDS, placed on an orbital shaker that provided gentle agitation.

Next, sections were washed 6 × 10 min in 0.1 M PBS followed by 90 min incubation in biotinylated secondary antibody (Donkey Anti-Rabbit IgG, 1:400; Jackson ImmunoResearch Laboratories, West Grove, PA, USA) diluted in 0.1 PBS-TX. The sections were then rinsed 4 × 10 min in 0.1 PBS and placed in avidin-biotin complex (ABC, 1:200; Vector Laboratories, Peterborough, UK) solution in PB for a further 90 min. Tissues were then washed 3 × 10 min in 0.1 M PB, followed by a brief rinse with distilled water. Fos-immunoreactive (Fos-ir) nuclei were visualised by reaction with nickel-intensified diaminobenzidine chromagen, Ni-DAB (DAB substrate kit; Vector Laboratories, Peterborough, UK) until yielding a defined blue/black stain. Sections were then rinsed in distilled water, mounted onto gelatine-coated glass slides and air-dried for 24 hours. Finally, sections were dehydrated through brief immersions in a series of graded ethanol concentrations (once in 70% and 95% and twice in 100%), cleared in Histoclear (National Diagnostics, Hull, UK), and cover-slipped in Vector-shield mounting medium (Vector Labs, Burlingame, CA) for visualisation and analysis. High-resolution digital photographs of representative sections were taken with an Olympus BX51 light microscope equipped with a digital camera (Olympus DP71) and Cell^F software. The final figures were prepared in Adobe Photoshop.

The SCN area was identified using previous anatomical verification (SCN:^80^) or local landmarks (e.g. optic chiasm, third ventricle) and a rat brain atlas^81^. The number of Fos-ir nuclei were double counted manually and unilaterally in each SCN section by a single observer blind to the light pulse received by each animal, using NIH ImageJ software (National Institutes of Health, Bethesda, MD). The counts were then averaged accordingly. No detectable Fos-ir staining was observed when c-fos primary antibody was omitted (data not shown).

### Melatonin Analysis

Plasma melatonin concentrations were quantified by radioimmunoassay (Stockgrand Ltd., University of Surrey, Guildford, UK), with a detection limit of 3 pg/ml. The intra-assay coefficients of variation (CV) for the low (mean ± SD, 9.3 ± 0.6 pg/ml), medium (32.8 ± 2.1 pg/ml), high (73.5 ± 1.8 pg/ml) and extra high (115.2 ± 4.6 pg/ml) pools were 6.5, 6.4, 2.4 and 4.0%, respectively. All samples were measured in duplicate in a single assay.

### Statistics

Data are expressed as the mean ± standard error of the mean (SEM). Statistical differences between parameters were evaluated by a repeated measures analysis of variance (ANOVA) followed by *post-hoc* pairwise comparisons by Bonferroni test. For independent groups, ANOVA followed by Bonferroni test or independent t-test were performed. Values of *p* < 0.05 were considered to be statistically significant. Non-Parametric Kruskal-Wallis followed by Mann-Whitney U test was performed on c-Fos data. All statistical analyses were performed with SPSS 15.0 software (SPSS Inc., Chicago, IL, USA).
